# Is IL-6 an appropriate target to treat spondyloarthritis patients refractory to anti-TNF therapy? a multicentre retrospective observational study

**DOI:** 10.1186/ar3766

**Published:** 2012-03-09

**Authors:** Fernando Kemta Lekpa, Cécile Poulain, Daniel Wendling, Martin Soubrier, Michel De Bandt, Jean Marie Berthelot, Philippe Gaudin, Eric Toussirot, Philippe Goupille, Thao Pham, Jérémie Sellam, Rémy Bruckert, Muriel Paul, Valérie Farrenq, Pascal Claudepierre

**Affiliations:** 1 Rheumatology Department, AP-HP, Henri Mondor University Hospital, 51 avenue du Mal de Lattre de Tassigny, 94010 Créteil, France; 2 Rheumatology Department, Jean Minjoz University Hospital, 2 boulevard Fleming, 25030 Besançon, France; 3 Rheumatology Department, Gabriel Montpied University Hospital, 58 rue Montalembert, 63000 Clermont-Ferrand, France; 4 Rheumatology Department, Robert Ballanger Hospital, boulevard Robert Ballanger, 93602 Aulnay sous Bois, France; 5 Rheumatology Department, Hôtel Dieu University Hospital, place Alexis Ricordeau, 44093 Nantes, France; 6 Rheumatology Department, University Hospital, Grenoble, Hôpital Sud, 19 avenue de Kimberley BP 185, 38130 Echirolles, France; 7 CIC-Biotherapy 506, St Jacques Hospital University Hospital, 2 boulevard Fleming, 25030 Besançon France; 8 Rheumatology Department, Trousseau University Hospital, 37044 Tours Cedex 1, France; 9 Rheumatology Department, Conception University Hospital, 147 boulevard Baille, 13385 Marseille, France; 10 Rheumatology Department, Saint-Antoine Hospital, Pierre et Marie Curie University, 184 rue du faubourg Saint Antoie, 75012 Paris, France; 11 INSERM Unit 955, AP-HP, Henri Mondor University Hospital, 51 avenue du Mal de Lattre de Tassigny, 94010 Créteil, France; 12 Pharmacy Department, AP-HP, Henri Mondor University Hospital, 51 avenue du Mal de Lattre de Tassigny, 94010 Créteil, France; 13 LIC EA4393, University Paris Est, 8 avenue du Général Sarrail, 94000 Créteil, France

## Abstract

**Introduction:**

The aim of this study was to evaluate, under real-life conditions, the safety and efficacy of tocilizumab in patients having failed anti-TNFα therapy for spondyloarthritis.

**Methods:**

French rheumatologists and internal-medicine practitioners registered on the Club Rhumatismes et Inflammations website were asked to report on patients given tocilizumab (4 or 8 mg/kg) to treat active disease meeting Assessment of SpondyloArthritis International Society (ASAS) criteria for axial or peripheral spondyloarthritis, after anti-TNFα treatment failure. Safety and efficacy after 3 and 6 months were assessed retrospectively using standardised questionnaires.

**Results:**

Data were obtained for 21 patients, 13 with axial spondyloarthritis (46% men; median age, 42 years; disease duration, 11 years; HLA-B27-positive, 92.3%) and eight with peripheral spondyloarthritis (25% men; median age, 40 years; disease duration, 10 years; HLA-B27-positive, 62.5%). No patients with axial disease had at least a 20 mm decrease in the BASDAI, nor a BASDAI50 response or major ASAS-endorsed disease activity score improvements after 3 or 6 months; an ASAS-endorsed disease activity score clinically important improvement was noted at month 3 in five of 13 patients and at month 6 in one of four patients. A good DAS28 response was achieved in four patients with peripheral disease, including one in EULAR remission at month 3. Four patients were still taking tocilizumab at month 6, including one in EULAR remission and one with a good DAS28 response. Tocilizumab was well tolerated, with no serious adverse events. Initially elevated acute-phase reactants declined during tocilizumab therapy.

**Conclusion:**

In patients having failed anti-TNFα therapy, tocilizumab decreased acute-phase reactants but failed to substantially improve axial spondyloarthritis and was inconsistently effective in peripheral spondyloarthritis.

## Introduction

The introduction of TNFα antagonists (anti-TNFα) has revolutionised the management of patients with spondyloarthritis (SpA). Randomised controlled trials demonstrated substantial efficacy of anti-TNFα therapy in ankylosing spondylitis (AS) [1-3] and psoriatic arthritis (PsA) [4-6], with high drug continuation rates [7,8]. Other treatment options are needed, however, for patients who experience primary or secondary failure of one or more anti-TNFα agents, as well as for those with contraindications to anti-TNFα therapy. Preliminary data suggested that other biologics licensed for rheumatoid arthritis (RA), such as rituximab [9,10] and abatacept [11-13], might hold promise for AS and PsA, but subsequent results in AS or undifferentiated SpA were disappointing [14-18].

In experimental studies, serum IL-6 levels were elevated in patients with AS and PsA and correlated with disease activity [19-22]. In addition, IL-6 was expressed in biopsies of sacroiliac joints from patients with AS, particularly those with recent-onset disease [23]. IL-6 blockade has been found effective in RA [24], another chronic inflammatory joint disease. These data suggest that IL-6 blockade may constitute a therapeutic option for SpA. One 1996 case report describes a good response in a patient with severe undifferentiated SpA given a murine monoclonal anti-IL-6 antibody combined with a monoclonal anti-CD4 antibody [25]. Since then, however, the few reports of patients with SpA given the anti-IL-6 agent tocilizumab indicated mixed effects [26-29].

Tocilizumab is a humanised monoclonal antibody to the human IL-6 receptor [30] and is licensed for use in active RA. Tocilizumab has been used in a few patients with AS or PsA who had failed treatment with the three anti-TNFα agents available in France and for whom no other treatment options existed. Under the auspices of the Club Rhumatismes et Inflammation (CRI), a section of the Société Française de Rhumatologie and a partnership with the French Society of Internal Medicine, our purpose was to evaluate the effectiveness and safety of tocilizumab therapy in patients with axial or peripheral SpA having failed anti-TNFα therapy, based on a retrospective analysis of data acquired under real-life conditions in France.

## Materials and methods

### Case ascertainment

All of the 1,400 French rheumatologists and internists belonging to the CRI were asked, through the CRI website [31], to report cases of active SpA treated with tocilizumab in their practice. Three successive monthly electronic newsletters were sent by email to collect observations. Reporting occurred from March 2011 to June 2011. All patients treated with tocilizumab had given their informed consent to this treatment, and the study has been approved by a French Institutional Review Board.

Among reported cases, we selected the patients who met at least the Assessment of SpondyloArthritis International Society (ASAS) criteria for axial SpA [32] or peripheral SpA [33] and who had active disease at baseline, defined as a Bath Ankylosing Spondylitis Disease Activity Index (BASDAI) ≥ 40 mm in the axial disease group and as a Disease Activity Score in 28 joints (DAS28) > 3.2 in the peripheral disease group. In addition, analysed patients had to have failed treatment with at least two anti-TNFα agents and to have received intravenous tocilizumab infusions in a dosage of 4 or 8 mg/kg every 4 weeks for at least 3 months.

### Assessment

For each patient, data were collected by the referent rheumatologist on a standardised case-report form available on the CRI website [31]. The following baseline data were recorded: medical history, former and current treatments, and disease classification criteria (ASAS axial SpA [32], ASAS peripheral SpA [33], modified New York criteria for AS [34], Amor's criteria [35], European Spondyloarthropathy Study Group criteria [36], and Classification Criteria for Psoriatic Arthritis [37]). Efficacy and safety data collected at baseline and at each tocilizumab infusion were the BASDAI, the Bath Ankylosing Spondylitis Functional Index (BASFI), the Assessment of SpondyloArthritis International Society-endorsed disease activity score (ASDAS), the DAS28, the pain score on a visual analogue scale (VAS), the patient's global assessment of disease activity, morning stiffness duration, tender joint count, swollen joint count (SJC), the presence of enthesitis or dactylitis, serum acute-phase reactants (C-reactive protein (CRP) and erythrocyte sedimentation rate (ESR)), concomitant treatments, and possible clinical or laboratory adverse events. Data were collected for the first 3 or 6 months of tocilizumab therapy.

### Data analysis

Completed case-report forms were sent to the coordinating centre (Henri Mondor Teaching Hospital). Patients meeting the study inclusion criteria were identified. Missing data on these patients were obtained by contacting the treatment centres.

Effectiveness was assessed based on data obtained at baseline (M0), after 3 months (M3), and after 6 months (M6). Patients with axial disease were classified as responders if they had at least a 20 mm decrease in the BASDAI or at least a 50% improvement in the BASDAI (BASDAI50 response) at M3 or M6, as proposed for assessment of efficacy of an anti-TNFα drug [38]; secondary efficacy endpoints were ASDAS major improvement (change by at least 2.0 points), ASDAS clinically important improvement (change by at least 1.1 points), percentage change in the VAS pain score, and percentage change in the BASFI. In the group with peripheral disease, the primary efficacy endpoint was a DAS28 response according to European League Against Rheumatism (EULAR) criteria for RA at M3 or M6. Secondary efficacy endpoints were the changes in the SJC and ASDAS, ASDAS major improvement, ASDAS clinically important improvement, BASDAI improvement, change in VAS pain score, presence of enthesitis, and corticosteroid-sparing effect.

Safety was assessed based on the rates of adverse events, serious adverse events, and infections, as well as on laboratory test results at each tocilizumab infusion.

## Results

### Baseline characteristics

We identified 21 patients meeting our inclusion criteria, 13 with axial disease and eight with peripheral disease, according to ASAS classification criteria; one of those patients has been reported elsewhere [29]. Only one reported case was not included, because of missing data.

Of the 13 patients with axial disease, nine also met Amor's criteria [35] and the European Spondyloarthropathy Study Group criteria [36]; eight of them had radiographic sacroiliitis and also met the modified New York criteria for AS [34]. All 13 patients had failed treatment with the three available anti-TNFα agents (Table 1 reports the main patient characteristics at baseline for this group). All 13 patients had morning stiffness of at least 30 minutes' duration; one patient also had synovitis at baseline (Patient 8), but suffered mainly from inflammatory neck and back pain, and thus met the ASAS classification criteria for axial SpA. Median age at diagnosis was 42 years (range 26 to 63 years) and median disease duration was 11 years (range 6 to 27 years). HLA-B27 was present in all the patients but one. In addition to anti-TNFα agents, five patients had also failed abatacept therapy and one had failed rituximab therapy. The tocilizumab dosage was 8 mg/kg in 12 patients and 4 mg/kg in one patient. All 13 patients were evaluated at M3 (after three tocilizumab infusions); four patients further continued the treatment and were also evaluated at M6 (after six tocilizumab infusions).

**Table 1:** Main baseline features of the 13 patients meeting ASAS criteria [30] for axial spondyloarthritis

	Patient 1	Patient 2	Patient 3	Patient 4	Patient 5	Patient 6	Patient 7
Type of SpA	AS	AS	AS	AS	AS	AS	AS
Sacroiliitis	Grade 4	Grade 4	Grade 3	Grade 4	Grade 3	Grade 3	Grade 4
HLA-B27	+	+	+	+	+	+	+
Gender	Female	Female	Female	Male	Male	Female	Male
Disease duration (years)	9	27	11	6	4	24	19
Main location of inflammatory pain	Back, neck, chest, peripheral enthesitis (Achilles tendon, knee)	Neck, chest, peripheral enthesitis^a^	Back, buttock, chest	Neck	Back, neck, buttock	Back, neck, chest	Back, neck, peripheral enthesitis (Achilles tendon)
Concomitant NSAIDs/steroids (dosage)	Yes/no	Yes/no	Yes/no	No/no	No/no	Yes/no	Yes/no
Concomitant DMARDs	No	No	No	Sulfasalazine	No	No	No
Previous biologics other than anti-TNF	Abatacept	Abatacept	Abatacept	No	No	No	No

	**Patient 8**	**Patient 9**	**Patient 10**	**Patient 11**	**Patient 12**	**Patient 13**	

Type of SpA	AS and PsA	uSpA	uSpA	uSpA	uSpA	uSpA	
Sacroiliitis	Grade 3	Grade 1	Grade 0	Grade 0	Grade 0	Grade 0	
HLA-B27	-	+	+	+	+	+	
Gender	Male	Female	Female	Male	Female	Male	
Disease duration (years)	15	11	9	25	6	12	
Main location of inflammatory pain	Back, neck, arthritis	Back, neck, buttock, chest	Back, buttock	Back, neck, buttock	Back, buttock, chest, peripheral enthesitis (Achilles tendon)	Back, neck	
Concomitant NSAIDs/steroids (dosage)	No/no	Yes/no	Yes/no	No/no	Yes/no	No/yes (25 mg/day)	
Concomitant DMARDs	No	No	No	No	No	No	
Previous biologics other than anti-TNF	Rituximab	Abatacept	Abatacept	No	No	No	

AS, ankylosing spondylitis meeting modified New York criteria [33]; ASAS, Assessment of SpondyloArthritis International Society; DMARD, disease-modifying antirheumatic drug; NSAID, nonsteroidal anti-inflammatory drug; PsA, psoriatic arthritis meeting Classification Criteria for Psoriatic Arthritis [36]; SpA, spondyloarthritis; uSpA, undifferentiated spondyloarthritis. ^a^Location unknown.

Of the eight patients with peripheral disease, seven also met Amor's criteria or the European Spondyloarthropathy Study Group criteria, four met modified New York criteria for AS, and two met the Classification Criteria for Psoriatic Arthritis. Seven patients had failed treatment with three anti-TNFα agents, and the remaining patient had failed treatment with two anti-TNFα agents (Table 2 presents the main patient characteristics at baseline for this group) One should note that, despite only one of the patients ha enthesitis, all patients had peripheral arthritis. Median age at diagnosis was 40 years (range 28 to 63 years) and median disease duration was 10 years (range 6 to 20 years). Of these eight patients, five were HLA-B27-positive. One patient had also failed abatacept therapy and another had failed anakinra therapy. The tocilizumab dosage was 8 mg/kg in all eight patients. All of the eight peripheral disease patients were evaluated at M3, and four patients were also evaluated at M6.

**Table 2:** Main baseline features in the eight patients meeting ASAS criteria [32] for peripheral spondyloarthritis

	Patient 14	Patient 15	Patient 16	Patient 17	Patient 18	Patient 19	Patient 20	Patient 21
Type of SpA	AS	AS	AS	AS	uSpA	uSpA	PsA	PsA
Sacroiliitis	Grade 2	Grade 3	Grade 3	Grade 3	Grade 0	Grade 1	Grade 0	Grade 0
HLA-B27	+	-	+	+	+	+	-	-
Gender	Male	Female	Male	Female	Female	Female	Female	Female
Disease duration (years)	8	15	19	6	10	10	10	20
Main articular and extraarticular symptoms	Coxitis, arthritis	Arthritis	Arthritis, uveitis	Peripheral enthesitis (Achilles tendon, knee, elbow), arthritis, chest pain	Arthritis	Arthritis, IBD, uveitis	Arthritis, dactylitis, psoriasis, uveitis	Arthritis, psoriasis
Concomitant NSAIDs/steroids (dosage)	Yes/no	No/yes (20 mg/day)	Yes/yes (5 mg/day)	Yes/yes (15 mg/day)	Yes/yes (8 mg/day)	No/yes (10 mg/day)	Yes/yes (20 mg/day)	Yes/yes (20 mg/day)
Concomitant DMARDs	Methotrexate	No	Methotrexate	Leflunomide	Methotrexate	Methotrexate	Methotrexate	Methotrexate
Previous biologics other than anti-TNF	No	Anakinra	No	Abatacept	No	No	No	No

AS, ankylosing spondylitis meeting modified New York criteria [33]; ASAS, Assessment of SpondyloArthritis International Society; DMARD, disease-modifying antirheumatic drug; IBD, inflammatory bowel disease; NSAID, nonsteroidal anti-inflammatory drug; PsA, psoriatic arthritis meeting Classification Criteria for Psoriatic Arthritis [36]; SpA, spondyloarthritis; uSpA, undifferentiated spondyloarthritis.

### Effectiveness

The effectiveness criteria at M0, M3, and M6 are reported in Table 3 for the axial disease group and in Table 4 for the peripheral disease group.

**Table 3:** Baseline and 3-month and 6-month tocilizumab therapy criteria in 13 patients with axial spondyloarthritis

	Pt 1	Pt 2	Pt 3	Pt 4	Pt 5	Pt 6	Pt 7	Pt 8	Pt 9	Pt 10	Pt 11	Pt 12	Pt 13
BASDAI, 0 to 100													
M0	78	90	72	42	77	63	80	48	49	59	48	72	57
M3 (change from M0; %)	+6	-19	+1	-15	+1	-1.6	+3.8	-2	+4	-5	+43	-10	-12
M6 (change from M0; %)	NA	NA	NA	-17	NA	NA	NA	NA	-6	-15	+17	NA	NA
ASDAS													
M0	4.87	3.35	3.24	3.37	5	5.36	3.8	2.98	2.68	2.41	2.94	3.2	4.12
M3 (change from M0; units)	-1.6	-0.8	-0.75	-0.04	-1.5	-1.7	-0.3	-0.24	-1.36	-0.43	-0.35	+0.02	-1.96
M6 (change from M0; units)	NA	NA	NA	-1.17	NA	NA	NA	NA	-1.02	-0.61	-0.23	NA	NA
BASFI, 0 to 100													
M0	86	95	63	32	72	MD	MD	59.5	50	57	59	28	36
M3 (change from M0; %)	-8	-8	+16	+3	+28	MD	MD	-6	+6	-7	+30	-29	+47
M6 (change from M0; %)	NA	NA	NA	+22	NA	MD	MD	NA	0	-7	-34	NA	NA
Pain VAS, 0 to 10													
M0	9	10	7	4	7	7	8	4	5	6	5	7	4
M3 (change from M0; units)	+0.5	-3	+1	0	0	-0.2	+1	+3	+1	-2	0	-1	0
M6 (change from M0; units)	NA	NA	NA	-1	NA	NA	NA	NA	+1	-2	+2	NA	NA
CRP (mg/dl)^a^													
M0	29	0	2	46	35	96	10	10.4	1	1	1.8	3	35.2
M3 (absolute value)	0.3	0	0	NA	3	4	1	< 5	NA	NA	NA	3	1
M6 (absolute value)	NA	NA	NA	6	NA	NA	NA	NA	0	1	0.6	NA	NA
ESR (mm)^a^													
M0	71	9	8	47	35	104	21	12	9	8	1	11	15
M3 (absolute value)	10	2	4	NA	2	2	1	3	NA	NA	NA	3	1
M6 (absolute value)	NA	NA	NA	5	NA	NA	NA	NA	4	1	1	NA	NA

BASDAI, Bath Ankylosing Spondylitis Disease Activity Index; ASDAS, Assessment of SpondyloArthritis International Society-endorsed disease activity score; BASFI, Bath Ankylosing Spondylitis Functional Index; VAS, visual analogue scale; CRP, C-reactive protein; ESR, erythrocyte sedimentation rate; M0, month 0 (baseline); M3, month 3; M6, month 6; MD, missing data; NA, not available; Pt, Patient. ^a^For CRP and ESR, the table shows only the values at baseline (M0) and at last follow-up (M3 or M6).

**Table 4:** Baseline and 3-month and 6-month tocilizumab therapy evaluation criteria in eight patients with peripheral spondyloarthritis

	Patient 14	Patient 15	Patient 16	Patient 17	Patient 18	Patient 19	Patient 20	Patient 21
DAS28								
M0	4.02	4.52	5	4.58	5.41	6.2	5.23	4.92
M3 (absolute value)	2.82	3.85	4.3	2.88	3.18	1.1	5.1	4.70
M3 EULAR response (yes/no)	Yes (good)	No	Yes (moderate)	Yes (good)	Yes (good)	Yes (remission)	No	No
M6 (absolute value)	1.95	4.01	5.34	3.12	NA	NA	NA	NA
M6 EULAR response (yes/no)	Yes (remission)	No	No	Yes (good)	NA	NA	NA	NA
ASDAS								
M0	2.41	4.03	MD	MD	MD	3.8	3	MD
M3 (absolute value)	1.50	2.64	MD	MD	MD	1.35	3.1	MD
M6 (absolute value)	1.03	2.79	MD	MD	MD	NA	NA	MD
SJC^a^								
M0	7	2	2	0	3	9	4	7
M3 (absolute value)	NA	NA	NA	NA	7	0	3	6
M6 (absolute value)	0	6	1	0	NA	NA	NA	NA
TJC^a^								
M0	7	6	5	5	0	15	7	12
M3 (absolute value)	NA	NA	NA	NA	0	0	6	11
M6 (absolute value)	2	4	3	8	NA	NA	NA	NA
BASDAI, 0 to 100								
M0	42	75	NA	79.4	67	48	4.7	NA
M3 (change from M0; %)	-31	-6.7	NA	-9	+6	NA	-2.1	NA
M6 (change from M0; %)	-59.5	-4.7	NA	-32	NA	NA	NA	NA
BASFI, 0 to 100								
M0	40	72	MD	MD	54	70	NA	NA
M3 (change from M0, %)	-17.5	-26	MD	MD	+44	NA	NA	NA
M6 (change from M0, %)	-12.5	-12.5	MD	MD	NA	NA	NA	NA
Pain VAS, 0 to 10								
M0	1.5	7	5	7	7	5	6	8
M3 (absolute value)	2	8	NA	6	8	1	6.5	6
M6 (absolute value)	1	9	6	7.5	NA	NA	NA	NA
PGA, 0 to 10								
M0	1	6	7	7.5	7	5.2	6.5	8
M3 (absolute value)	3.5	6	MD	MD	7	1	6.5	8
M6 (absolute value)	1.2	6	8	MD	NA	NA	NA	NA
CRP (mg/dl)^a^								
M0	20.2	17	119	12	112.3	65	4	1
M3 (absolute value)	NA	NA	NA	NA	9.5	1	5	1
M6 (absolute value)	0.4	0.8	128	1	NA	NA	NA	NA
ESR (mm)^a^								
M0	39	22	67	26	103	60	27	7
M3 (absolute value)	NA	NA	NA	NA	8	4	7	8
M6 (absolute value)	5	7	70	2	NA	NA	NA	NA

ASDAS, Assessment of SpondyloArthritis International Society endorsed disease activity score; BASDAI, Bath Ankylosing Spondylitis Disease Activity Index; BASFI, Bath Ankylosing Spondylitis Functional Index; CRP, C-reactive protein; DAS28, Disease Activity Score in 28 joints; ESR, erythrocyte sedimentation rate; EULAR, European League Against Rheumatism; M0, month 0 (baseline); M3, month 3; M6, month 6; NA, not available; MD, missing data; PGA, patient's global assessment of disease activity; SJC, swollen joint count; TJC, tender joint count; VAS, visual analogue scale. ^a^For SJC, TJC, CRP, and ESR, the table shows only the values at baseline (M0) and at last follow-up (M3 or M6).

In the group of patients with axial disease, no patient at M3 had achieved at least a 20 mm decrease in BASDAI, nor a BASDAI50 response (Table 3). Of the 13 patients, five had an ASDAS clinically important improvement but none had an ASDAS major improvement. The VAS pain score and the BASFI were unchanged overall. Nevertheless, tocilizumab therapy was continued for three additional months in four patients. At M6, none of these four patients had achieved at least a 20 mm decrease in BASDAI or a BASDAI50 response, one patient had an ASDAS clinically important improvement, and no patients had an ASDAS major improvement. A single patient had a 20% BASFI decrease.

Of the eight patients with AS in the axial disease group (Patients 1 to 8), none had at least a 20 mm decrease in BASDAI or a BASDAI50 response at M3, three had an ASDAS clinically important improvement, none had an ASDAS major improvement, and only one patient continued tocilizumab therapy until M6, when the response was moderate.

Of the five patients (out of the total 21) with a very high disease activity at baseline according to the ASDAS (ASDAS > 3.5), four reached an ASDAS clinically important improvement.

In the group with peripheral disease, the DAS28 at baseline was ≥ 4 in all eight patients. At M3, four patients had a good DAS28 response, including one who met EULAR remission criteria, and another patient had a moderate DAS28 response. The remaining three patients had an insufficient response. At last follow-up, the SJC had decreased in five of the seven patients with joint swelling at baseline, including two patients who had no clinical synovitis; in the remaining two patients, the SJC had increased. At least a 20% SJC decrease was noted in four patients. The ASDAS was available at M3 for four patients and showed a clinically important improvement in one patient and a major improvement in one patient. These two patients were those that had a very high baseline disease activity according to the ASDAS (> 3.5). No patient had a BASDAI50 response. The VAS pain score decreased slightly in two patients and increased in four patients. Of the four patients treated for 6 months, two had a good DAS28 response at M6, including one who was in remission, and two had an insufficient DAS28 response. The ASDAS was available at M6 in two patients and showed a clinically important improvement with no major improvement in both. At M6, the VAS pain score was increased in three of four patients and diminished by 0.5 point in one patient.

Of the eight patients with peripheral disease, seven were taking glucocorticoid therapy at baseline. The glucocorticoid dosage was increased during tocilizumab therapy in three patients, left unchanged in two patients, and decreased in two patients. Two patients received three and two local corticosteroid injections, respectively.

Of the four patients with peripheral AS (Patients 14 to 17), two had a good DAS28 response and one had a moderate DAS 28 response. Neither of the two patients with PsA (Patients 20 and 21) achieved a clinically relevant improvement.

### Acute-phase reactants

Of the seven axial disease patients with CRP elevation at baseline, six had normal CRP values at M3; the M3 CRP level was missing in the remaining patient. Similarly, the ESR decreased substantially in the patients with ESR elevation at baseline (Table 3).

Of the six peripheral disease patients with CRP elevation at baseline, two patients had normal CRP values at M3 and four patients at M6. The CRP level had increased in one patient at M6. The ESR decreased substantially, except in the patient whose CRP value had increased by M6.

### Safety

No serious adverse events occurred in either group. The following adverse events were reported: axillary abscess (Patient 1), toe blister (Patient 1), allergic reaction (Patient 2), oral ulcers (Patients 14 and 17), nasopharyngitis (Patient 15), Q fever (Patient 16), vasospasm of the extremities (Patient 17), liver enzyme elevation < 3N (Patient 8), and cholesterol and triglyceride elevation (Patient 8).

Flares of anterior uveitis occurred in two patients (Patients 7 and 16) with previous episodes of uveitis, and *de novo *acute anterior uveitis occurred in one patient (Patient 5). A flare of psoriasis also occurred in Patient 16, who had a previous history of psoriasis.

## Discussion

We obtained detailed descriptions of 3-month and 6-month outcomes of tocilizumab therapy in 21 patients with SpA who had failed anti-TNFα therapy. In the 13 patients meeting the ASAS criteria for axial SpA [32], tocilizumab therapy produced no significant improvement based on BASDAI variation or ASDAS major improvement at M3 (*n *= 13) or M6 (*n *= 4). An ASDAS clinically important improvement was achieved in five patients at M3 and in one patient at M6. In the eight patients meeting the ASAS criteria for peripheral SpA [33], the effects of tocilizumab therapy were mixed: at M3, four patients had a good DAS28 response, including one in EULAR remission; and four patients were still receiving tocilizumab at M6, including one in EULAR remission and one with a good DAS28 response. Globally, tocilizumab was safe in both groups, with few adverse events and no serious adverse events.

Tocilizumab provided no clinically relevant effects in the patients with axial disease. The effects were mixed in the group with peripheral disease. Tocilizumab was ineffective in both the patients with axial AS and in those with axial undifferentiated SpA. The numbers of patients with peripheral SpA were too small for subgroup analyses by diagnosis; however, the good M6 response in the two patients with peripheral AS is worth noting.

We found four previous case reports of tocilizumab therapy for SpA published in 2009 and 2010 [26-29]. The effects in these reports were mixed, in keeping with our findings. In the two patients with peripheral SpA, tocilizumab induced clinical improvements [26,27]. The other two patients had axial SpA; among these, one patient benefited clinically but had no improvements in spinal magnetic resonance imaging findings after 18 weeks [28], and the other had no clinical improvements [29]. Two preliminary reports in groups of patients have been published: one was a study of 18 patients with axial disease given tocilizumab for a median of 6.5 months, which showed improvements in a single patient [39]; the other study showed no meaningful clinical effects after three to six tocilizumab infusions in five patients with axial SpA [40]. Taken together, our study findings and previously published data fail to indicate substantial effectiveness of tocilizumab in axial SpA refractory to anti-TNFα therapy and suggest limited effectiveness in peripheral forms. These results may seem surprising, given the available evidence that IL-6 plays a significant pathogenic role in SpA [19-23]. In an animal model of TNF-mediated bilateral sacroiliitis, however, IL-6 was not a crucial regulator of the disease process [41]. These findings point to substantial differences in the mechanisms underlying RA and SpA, which translate into differences in the effectiveness of pharmacotherapeutic agents. Several other drugs known to be effective in RA have also failed to improve patients with SpA, including methotrexate, rituximab [14-16], abatacept [17,18], and anakinra [42-44].

The absence of meaningful clinical improvements in our study contrasted with decreases in acute-phase reactants during tocilizumab therapy. These decreases indicate that IL-6 blockade was effective in our patients. Our results demonstrate that the markers for systemic inflammation can return to normal despite no clinical improvement. Consequently, ESR and CRP values cannot be used as isolated surrogate markers for efficacy in SpA patients, even for those with elevated ESR and CRP values at baseline. On the other hand, incorporating ESR or CRP into composite scores such as the ASDAS can improve the assessment of patients with SpA, and perhaps improve the selection of candidate patients for other biologics after several failures. In line with this hypothesis we can note that, within axial patients with a very high disease activity at baseline according to the ASDAS (ASDAS > 3.5), we observed four out of five (80%) patients with an ASDAS clinically important improvement.

Our study has several limitations. Firstly, we should highlight the retrospective design and the limited number of patients. Our purpose was not to conduct a therapeutic trial, however, but instead was to learn from the patients who had been treated under real-life conditions in France. Case ascertainment was incomplete, because not all rheumatologists and internists in France belong to the CRI; on the other hand, those who do belong to the CRI are used to providing detailed case reports - indeed, we excluded only one case because of missing data. We selected patients fulfilling recent ASAS criteria for axial SpA [32] or for peripheral SpA [33], and we analysed these two groups separately because they are clearly distinguished by the criteria.

Secondly, BASDAI alone is not specific enough to evaluate the inflammatory activity of axial SpA in these highly refractory patients. However, the BASDAI and expert opinion are the criteria recommended in France to start biologics after failure of non steroidal anti-inflammatory drugs. There is clearly a need to find new, more stringent, criteria for refractory patients failing treatment with at least two biologics. The ASDAS is a good candidate for these new criteria. It should be argued that some of our patients might actually have other causes of back pain than SpA; however, they all had significant morning stiffness and more than one-half of the axial patients had an increased baseline CRP level, suggesting that SpA itself was still active in those patients. In addition, doses and methods of administration of tocilizumab are those used in RA, a disease with different pathogenesis. Further therapeutic approaches different from those used for RA are needed, reaching as far as research on the new pathogenesis hypothesis in SpA.

## Conclusion

Despite a significant decrease of acute-phase reactants, IL-6 blockade did not improve the clinical status of patients with axial SpA who had previously failed anti-TNFα therapy. Tocilizumab does not seem to hold promise for the management of axial SpA refractory to anti-TNFα agents, although only the ongoing randomised controlled trials with this drug in AS could confirm this supposition. There is a need for studies of other drugs [45], different from those used in RA, as well as for continuing research into the pathogenesis of SpA.

## Abbreviations

anti-TNFα: TNFα antagonists; AS: ankylosing spondylitis; ASAS: Assessment of SpondyloArthritis International Society; ASDAS: Assessment of SpondyloArthritis International Society-endorsed disease activity score; BASDAI: Bath Ankylosing Spondylitis Disease Activity Index; BASDAI50: 50% improvement in the Bath Ankylosing Spondylitis Disease Activity Index; BASFI: Bath Ankylosing Spondylitis Functional Index; CRI: Club Rhumatismes et Inflammation; CRP: C-reactive protein; DAS28: disease activity score in 28 joints; ESR: erythrocyte sedimentation rate; EULAR: European League Against Rheumatism; IL: interleukin; M0, month 0 (baseline); M3: month 3; M6: month 6; PsA: psoriatic arthritis; RA: rheumatoid arthritis; SJC: swollen joint count; SpA: spondyloarthritis; TNF: tumour necrosis factor; VAS: visual analogue scale.

## Competing interests

DW has received consulting fees, speaking fees and/or honoraria from Abbott, Amgen, Wyeth-Pfizer, Roche, Chugai, Bristol-Myers Squibb, and Schering-Plough (< $10,000 each), and was investigator for Roche Chugai, Sanofi Aventis, and Novartis. MS has received consulting fees, speaking fees, and/or honoraria from Roche, Pfizer (Wyeth), MSD (Schering Plough), BMS, and Abbott. JMB has received consulting fees, speaking fees, and/or honoraria from Roche (< $10,000 each). PGa has received consulting fees, speaking fees and/or honoraria from Abbott, Wyeth-Pfizer, and Roche Chugai (< $10,000 each). PGo has received consulting fees, speaking fees and/or honoraria from Abbott, Wyeth-Pfizer, Roche, Chugai, Bristol-Myers Squibb, Schering-Plough, and MSD (< $10,000 each), and was investigator for Abbott, Wyeth-Pfizer, Roche Chugai, and Bristol-Myers Squibb. TP has received consulting fees, speaking fees and/or honoraria from Abbott, Amgen, Wyeth-Pfizer, Roche, Chugai, Bristol-Myers Squibb, and Schering-Plough (< $10,000 each), and was investigator for Roche Chugai, Sanofi Aventis, and Novartis. JS has received consulting fees, speaking fees, and/or honoraria from Pfizer (Wyeth), MSD (Schering Plough), and Roche France (< $10,000 each). PC has received consulting fees, speaking fees and/or honoraria from Abbott, Wyeth-Pfizer, Roche, Chugai, Bristol-Myers Squibb, Schering-Plough, and UCB (< $10,000 each), and was investigator for Roche Chugai, Sanofi Aventis, Abbott, Wyeth-Pfizer, and BMS (< $10,000 each). FKL, CP, MDB, ET, RB, MP, and VF declare that they have no competing interests.

## Authors' contributions

All authors were involved in drafting the article or revising it critically for important intellectual content, and all authors approved the final version to be published. PC had full access to all of the data in the study and takes responsibility for the integrity of the data and the accuracy of the data analysis. FKL, CP, DW, TP, JS, MP, and PC were involved in the study conception and design. FKL, CP, DW, MS, MDB, JMB, PGa, ET, PGo, TP, JS, RB, MP, VF, and PC were involved in the acquisition of data. FKL, CP, and PC were involved in the analysis and interpretation of data.
